# The real-life number of neonatal doses of Bacille Calmette-Guérin vaccine in a 20-dose vial

**DOI:** 10.1080/16549716.2017.1267964

**Published:** 2017-01-27

**Authors:** Frederik Schaltz-Buchholzer, Hannah Nørtoft Frankel, Christine Stabell Benn

**Affiliations:** ^a^ Research Center for Vitamins and Vaccines, Statens Serum Institut, Copenhagen, Denmark; ^b^ OPEN, Odense Patient data Explorative Network, Odense University Hospital/Institute of Clinical Research, University of Southern Denmark, Odense, Denmark; ^c^ Institute of Clinical Research, University of Southern Denmark, Odense, Denmark

**Keywords:** BCG vaccine, wastage, vial size, cost-effectiveness, infants

## Abstract

**Background**: Reducing vaccine wastage is important. Bacille Calmette-Guérin (BCG) vaccine is produced in vials of 20 infant doses. The reconstituted vaccine is discarded after 4–6 hours. Therefore, to reduce vaccine wastage, a 20-dose vial of BCG is often only opened if at least 10–12 infants are present, jeopardising BCG vaccination coverage and timely vaccination. We observed that nurses were not able to withdraw 20 doses from the vials and aimed to quantify how many doses could be obtained from these vials by experienced nurses under real-life circumstances.

**Methods**: At the maternity ward of the national hospital in Guinea-Bissau, since 2002 the same two nurses have been vaccinating all eligible children with BCG before discharge. During a month in 2015, within a randomised trial comparing BCG-Denmark and BCG-Russia, we registered how many doses the nurses were able to withdraw from the two types of vaccine vials.

**Results**: The median number of doses which it was possible to withdraw from the vials was 13 (range 11–17): 13 (11–16) for BCG-Denmark (based on 39 vials) and 15 (12–17) for BCG-Russia (based on 29 vials).

**Conclusions**: In real life, experienced nurses could only obtain 13–15 doses from the 20-dose vials. Thus, vaccine wastage is much lower than assumed. Adjusting practice to the real-life number of doses would immediately suggest vials should be opened if 7 rather than 10 infants are present. As other studies have indicated that BCG may have beneficial non-specific effects on overall mortality, the potential gain by opening a 20-dose vial even for one child may be considerable.

## Background

Bacille Calmette-Guérin (BCG) vaccine, the live attenuated vaccine against tuberculosis, is provided as a vial of freeze-dried vaccine together with a vial containing 1 ml solvent. According to the manufacturers’ specifications, one vial of reconstituted vaccine contains 1 ml, corresponding to 10 doses for adults and children aged 12 months and over (0.1 ml) or 20 doses for infants less than 12 months (0.05 ml). The reconstituted vaccine should be discarded after 4–6 hours [1,2].

Discarding remaining BCG vaccine after completion of a vaccine session, so-called ‘open vial wastage’, is an important source of wastage. Though the World Health Organization (WHO) states that ‘it is always recommended to open a vial of vaccine for one infant or a small number of infants’ [3], national vaccination programs are increasingly instructed to avoid wastage. Countries receiving support from the Global Alliance for Vaccines and Immunisations have been requested to reduce their wastage rates to 15% for 20-dose formulations [3].

One way to reduce open vial wastage is to increase the number of children vaccinated at each session [3]. Indeed, this is what is happening; in Guinea-Bissau and other low-income countries, a vial of BCG is not opened unless there are at least 10–12 infants eligible for BCG vaccination present. In practical terms, this is implemented by health centres having only one or two BCG vaccination days (BCG-days) per week or even per month. Mothers of children presenting on other days are told to come back on a BCG-day. If fewer than the required number of children are present on a given BCG-day, all children are told to come back on the next BCG-day. Such local policies cause unnecessary delays in vaccination and are a threat to obtaining full BCG vaccination coverage.

In order to calculate correct wastage rates, it is important to know the real-life number of doses in a 20-dose vial. In Guinea-Bissau, the Bandim Health Project (BHP) has conducted several randomised controlled trials (RCT) using BCG vaccine. For this purpose, the BHP has implemented BCG vaccination at the maternity ward of the national hospital, and since 2002, the same two skilled BCG vaccinators have vaccinated all neonates eligible for BCG vaccine. We used this set-up to test how many doses two experienced vaccinators could obtain from two different types of 20-dose BCG vaccine preparations: BCG-Denmark and BCG-Russia, both pre-qualified vaccines used interchangeably by UNICEF.

## Methods

In Guinea-Bissau, approximately 6,500 children/year are delivered at the maternity ward of the national hospital.

Within an ongoing trial, BCG-Denmark (Statens Serum Institut, Copenhagen, Denmark) and BCG-Russia (Serum Institute of India, Pune, India) are compared for their effect on overall morbidity within the first 6 weeks of life. Healthy neonates delivered at the maternity ward are randomised 1:1 to the two vaccines at discharge, with an average enrolment rate of 14 children per day. In addition, a smaller RCT at the hospital’s neonatal intensive care unit randomises neonates 1:1 to receive BCG-Denmark either at admission or at discharge. The average inclusion rate for this trial is three per day. Furthermore, BCG may be given to infants born at the hospital who did not participate in an RCT (due to lack of consent or severe malformations).

One of the two vaccinators provide all vaccinations on a given day. Vaccines are administered intradermally using a SoloShot Mini auto-disabling 0.05 ml syringe (Becton Dickinson, NJ, USA). After each vaccination, the vaccinator stores the used syringes in a safe container, one for BCG-Denmark and one for BCG-Russia.

The present study took place from 12 October–9 November 2015. At the end of each vaccination session, the vaccinator counted the used syringes for each vial of BCG opened. Subsequently, the vaccinator drew up any excess vaccine doses left in each vial, using a new 0.05 ml BCG syringe for each dose, until no further full 0.05 ml dose could be drawn. For each vial, the vaccinator noted the number of doses used during the vaccination session and the number of doses subsequently drawn from the vial. All procedures were supervised on a daily basis by a BHP staff member.

Ethical approval for the BCG trial was obtained from the Guinean Ethical Committee; the Danish ethical committee gave its consultative approval. No permission was necessary for the present study, which did not involve any study participants.

With 28 vials of each vaccine, the study would have 95% power to detect if the vaccine vials in fact contained 19 rather than 20 doses, assuming an SD of 1.6 and with a one-sided alpha of 0.05.

All statistical analyses were done as linear regression analyses in Stata 12.1.

## Results

**Figure 1.**
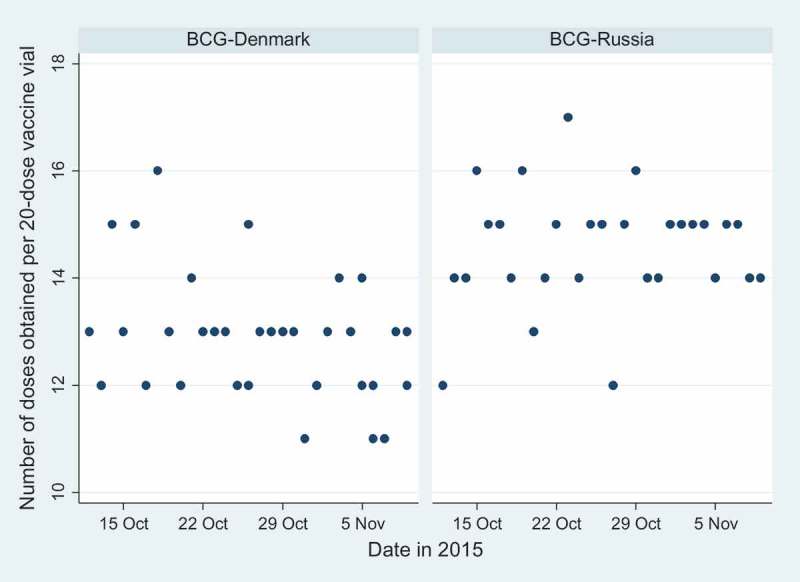
Number of doses obtained from 20-dose vials of BCG-Denmark and BCG-Russia, respectively. Guinea-Bissau, October–November 2015.

During 29 days, the two vaccinators opened 68 BCG vaccine vials, 39 (57%) BCG-Denmark and 29 (43%) BCG-Russia (see Supplementary Table). The median number of doses obtained was 13 (range 11–17): 13 (range 11–16) for BCG-Denmark and 15 (range 12–17) for BCG-Russia. More doses were obtained from the BCG-Russia vials than the BCG-Denmark vials (*p* < 0.00001, Figure 1). There was no significant difference in the number of doses obtained by the two vaccinators, neither overall nor by vaccine type (data not shown). The number of doses obtained per vial did not change over the study period, neither overall, by vaccine type (Figure 1), nor by vaccinator (data not shown).

## Discussion

We found that experienced vaccinators were able to retrieve significantly fewer infant vaccine doses from a 20-dose BCG vial than suggested by the manufacturers. The overall median number of doses obtained per vial was 13. There were more doses in the BCG-Russia vials than in the BCG-Denmark vials. Only once was it possible to extract a maximum of 17 doses.

Strengths of the study include the use of two different commercial vaccine preparations as well as two highly experienced vaccinators, both with more than 10 years of experience. The fact that there were no differences between the two vaccinators in the number of doses obtained for each of the two preparations of BCG vaccine suggests our findings were not due to operator-dependent artefacts but product-related. A limitation of the study is that the results are not directly generalisable to other settings with less experienced staff. Less experienced vaccinators would likely have a larger loss of vaccine due to poorer technique with respect to reconstituting the vaccine and withdrawing doses. Thus, we are confident that in real-life situations the 20-dose vials contain at best only around 13 (BCG-Denmark) and 15 (BCG-Russia) doses, respectively.

Common sense predicts that it would be implausible to obtain 20 doses of 0.05 ml each, when combining freeze-dried vaccine substance with 1 ml solvent, considering the several sources of wastage: first, loss in solvent vial (remaining liquid); second, dead space in needle used to reconstitute the vaccine; third, loss in vial with reconstituted vaccine; fourth, dead space in needle for injection; fifth, loss in de-airing of the syringe before injection [3]. Vaccine manufacturers are expected to overfill vials to compensate for vaccine wastage attributable to dead space in syringes to ensure that the number of doses indicated on the label can be drawn from the vial [3]. Based on our figures, there is too little BCG solvent to compensate for the wastage caused by syringes and remaining liquid in the solvent and BCG vials.

The results are very important because using the correct denominator to calculate open vial wastage rates for BCG could help provide realistic wastage targets. With a request to keep wastage for 20-dose vials at 15% [3], an average of 17 infants should be vaccinated with each vial to reach the target. This is not feasible as our team could only once obtain 17 doses from a vial. If it were acknowledged that the vials rather contain 13 doses, the 15% wastage target would be reached with 11 infants rather than 17 infants. This would imply an adjustment to the current practice in which opening a vial should happen when 7–8 rather than 10–12 infants are present.

This change in perception would immediately alleviate pressure on health workers, no longer having to restrict BCG vial opening as fiercely, with important consequences for BCG vaccination coverage, and for the mothers, who often come several times in vain before getting their child vaccinated. For instance, in 2010 in rural Guinea-Bissau, only 38% were BCG-vaccinated by 1 month of age, yet 25% of the BCG-unvaccinated children had been in contact with a health facility; many of their mothers reported having been told to come back another day for vaccination. Had the children been vaccinated at that contact, the BCG vaccination coverage by 1 month would have soared to 54% rather than 38% [4].

Timely BCG vaccination is particularly important because BCG vaccine provided at birth has been shown in RCTs to reduce neonatal mortality by more than 40% [5,6]. The gain from opening a vial of BCG just for one child may thus be considerable. The WHO advocates BCG at birth, but it is often given with large delays because of pressure to reduce wastage. It has been suggested that using vials with fewer doses would reduce wastage, but it has also been shown that the cost of such vials is not substantially lower, and decreased availability of vaccines and increased transport and storage facility costs would outweigh or even exceed the potential savings [7]. Thus, we recommend that a 20-dose vial of BCG vaccine be reclassified as a ‘1-dose vial with elastic’, which is opened for a single child, but can be extended to vaccinate up to 13–15 children.

## Conclusion

We found that in real life, experienced nurses could only obtain 13-15 doses from 20-dose BCG vials. Currently, to reduce vaccine wastage, in many low-income countries a BCG vial is not opened for less than 10-12 infants. Adjusting wastage-reducing practice to the real-life number of doses would immediately suggest vials be opened if 7 infants are present. As BCG may have beneficial non-specific effects, the potential gain by opening a vial even for one child may be considerable.

## Supplementary Material

GHA_33432_Stabell_Benn_Supplementary_table.docx

## Paper context

BCG vaccine comes in vials of 20 infant doses, which should be discarded 4–6 hours after opening. To reduce wastage, many countries only open a vial if 10–12 eligible infants are present. We demonstrated that in real life trained nurses only obtain 13 doses from the vials. This should lead to immediate adjustment of wastage-reducing practices. As BCG vaccine may have beneficial non-specific effects, opening a vial for one child may be highly cost-effective.

## References

[CIT0001] (2016). BCG Denmark product summary.

[CIT0002] (2016). BCG Russia product summary.

[CIT0003] WHO (2005). Monitoring vaccine wastage at country level. Guidelines for programme managers.

[CIT0004] Thysen SM, Byberg S, Pedersen M (2014). BCG coverage and barriers to BCG vaccination in Guinea-Bissau: an observational study. BMC Public Health.

[CIT0005] Aaby P, Roth A, Ravn H (2011). Randomized trial of BCG vaccination at birth to low-birth-weight children: beneficial nonspecific effects in the neonatal period?. J Infect Dis.

[CIT0006] Biering-Sørensen S, Aaby P, Napirna BM (2012). Small randomized trial among low-birth-weight children receiving bacillus Calmette-Guérin vaccination at first health center contact. Pediatr Infect Dis J.

[CIT0007] Assi T-M, Brown ST, Djibo A (2011). Impact of changing the measles vaccine vial size on Niger’s vaccine supply chain: a computational model. BMC Public Health.

