# Supplementary material for: The real-life number of neonatal doses of Bacille Calmette-Guérin vaccine in a 20-dose vial
Source: Glob Health Action. 2017 Jan 27;10(1):1267964. doi: 10.1080/16549716.2017.1267964 (PMC5328326; doi:10.1080/16549716.2017.1267964)
Supplement: GHA_33432_Stabell_Benn_Supplementary_table.docx [file zgha_a_1267964_sm0362.docx]

**Supplementary Table. Number of doses used and left in 20-dose vials of BCG-Denmark and BCG-Russia at the national hospital’s maternity ward. Guinea-Bissau, 12 October-9 November 2015.**

| **BCG-Denmark** | | | | | **BCG-Russia** | | | | |
| --- | --- | --- | --- | --- | --- | --- | --- | --- | --- |
| **Date** | **Vaccinator** | **Doses used** | **Doses left** | **Total** | **Date** | **Vaccinator** | **Doses used** | **Doses left** | **Total** |
| 12/10 | 1 | 13 | 0 | 13 | 12/10 | 1 | 9 | 3 | 12 |
| 12/10 | 1 | 12 | 1 | 13 |  |  |  |  |  |
| 13/10 | 1 | 12 | 0 | 12 | 13/10 | 1 | 14 | 0 | 14 |
| 13/10 | 1 | 3 | 9 | 12 |  |  |  |  |  |
| 14/10 | 1 | 14 | 1 | 15 | 14/10 | 1 | 12 | 2 | 14 |
| 15/10 | 1 | 13 | 0 | 13 | 15/10 | 1 | 6 | 10 | 16 |
| 16/10 | 1 | 12 | 3 | 15 | 16/10 | 1 | 8 | 7 | 15 |
| 17/10 | 1 | 2 | 10 | 12 | 17/10 | 1 | 4 | 11 | 15 |
| 18/10 | 1 | 10 | 6 | 16 | 18/10 | 1 | 12 | 2 | 14 |
| 19/10 | 2 | 12 | 1 | 13 | 19/10 | 2 | 9 | 7 | 16 |
| 19/10 | 2 | 6 | 7 | 13 |  |  |  |  |  |
| 20/10 | 2 | 10 | 2 | 12 | 20/10 | 2 | 8 | 5 | 13 |
| 21/10 | 1 | 11 | 3 | 14 | 21/10 | 1 | 8 | 6 | 14 |
| 22/10 | 1 | 12 | 1 | 13 | 22/10 | 1 | 6 | 9 | 15 |
| 23/10 | 1 | 7 | 6 | 13 | 23/10 | 1 | 5 | 12 | 17 |
| 24/10 | 2 | 9 | 4 | 13 | 24/10 | 2 | 12 | 2 | 14 |
| 25/10 | 2 | 11 | 1 | 12 | 25/10 | 2 | 10 | 5 | 15 |
| 25/10 | 2 | 1 | 11 | 12 |  |  |  |  |  |
| 26/10 | 1 | 12 | 0 | 12 | 26/10 | 1 | 12 | 3 | 15 |
| 26/10 | 1 | 9 | 6 | 15 |  |  |  |  |  |
| 27/10 | 1 | 6 | 7 | 13 | 27/10 | 1 | 7 | 5 | 12 |
| 28/10 | 1 | 8 | 5 | 13 | 28/10 | 1 | 7 | 8 | 15 |
| 29/10 | 1 | 6 | 7 | 13 | 29/10 | 1 | 7 | 9 | 16 |
| 30/10 | 1 | 13 | 0 | 13 | 30/10 | 1 | 14 | 0 | 14 |
| 30/10 | 1 | 1 | 12 | 13 |  |  |  |  |  |
| 31/10 | 1 | 11 | 0 | 11 | 31/10 | 1 | 12 | 2 | 14 |
| 01/11 | 1 | 8 | 4 | 12 | 01/11 | 1 | 9 | 6 | 15 |
| 02/11 | 2 | 11 | 2 | 13 | 02/11 | 2 | 11 | 4 | 15 |
| 03/11 | 1 | 13 | 1 | 14 | 03/11 | 1 | 8 | 7 | 15 |
| 04/11 | 1 | 13 | 0 | 13 | 04/11 | 1 | 14 | 1 | 15 |
| 04/11 | 1 | 4 | 9 | 13 |  |  |  |  |  |
| 05/11 | 1 | 14 | 0 | 14 | 05/11 | 1 | 13 | 1 | 14 |
| 05/11 | 1 | 1 | 11 | 12 |  |  |  |  |  |
| 06/11 | 1 | 12 | 0 | 12 | 06/11 | 1 | 12 | 3 | 15 |
| 06/11 | 1 | 2 | 9 | 11 |  |  |  |  |  |
| 07/11 | 2 | 6 | 5 | 11 | 07/11 | 2 | 5 | 10 | 15 |
| 08/11 | 2 | 7 | 6 | 13 | 08/11 | 2 | 7 | 7 | 14 |
| 09/11 | 1 | 13 | 0 | 13 | 09/11 | 1 | 9 | 5 | 14 |
| 09/11 | 1 | 5 | 7 | 12 |  |  |  |  |  |
